# Characterisation of soil micro-topography using a depth camera

**DOI:** 10.1016/j.mex.2020.101144

**Published:** 2020-11-12

**Authors:** Laura Grundy, Chandra Ghimire, Val Snow

**Affiliations:** AgResearch, Private Bag 4749, Christchurch 8140, New Zealand

**Keywords:** Structured light, Erosion, Runoff, Soil surface, Surface roughness

## Abstract

Soil surface roughness controls how water ponds on and flows over soil surfaces. It is a crucial parameter for erosion and runoff studies. Surface roughness has traditionally been measured using manual techniques that are simple but laborious. Newer technologies have been proposed that are less laborious but require expensive equipment and considerable expertise. New depth-camera technologies might provide a useful alternative. We tested the ability of one such camera to measure soil surface roughness. The camera's accuracy was good but decreased with camera-soil distance (0.3% at 750 mm and 0.5% at 1500 mm) however it was very precise (< 0.5 mm for elevation and < 0.05 mm for random roughness). Similarly, the error of the surface area estimation increased with camera-soil distance (0.56% at 750 mm and 2.3% at 1500 mm). We describe the workflow to produce high-resolution digital elevation models from initial images and describe the conditions under which the camera will not work well (e.g. extremes of lighting conditions, inappropriate post-processing options). The camera was reliable, required little in the way of additional technology and was practical to use in the field. We propose that depth cameras are a simple and inexpensive alternative to existing techniques.

• We tested a commercially-available 3D depth camera.
• The camera gave highly accurate and precise soil surface measurements.
• The camera provides an inexpensive alternative to existing techniques.

## Graphical abstract

**Figure:**
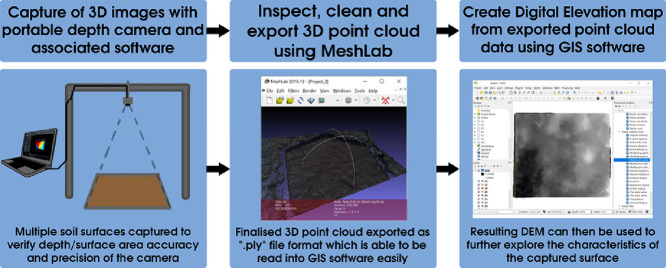

**Specifications table**

**Table:** 

Subject area:	Computer Science
More specific subject area:	Environmental Science
Method name:	Characterisation of soil micro-topography using a depth camera
Name and reference of original method:	J. Geng, Structured-light 3D surface imaging: a tutorial, Adv. Opt. Photon. 3(2) (2011) 128–160.
Resource availability:	N/A

## Introduction

Surface microtopography significantly controls surface water storage, infiltration, overland flow, and ultimately soil particle detachment and erosion at the plot scale [1], [2], [3], [4], [5]. Moreover, surface roughness is a key input parameter for physically-based water infiltration and storage models [6,7] and soil erosion models [8,9]. In such models, hydrological representation of the micro-topography is generally simplified using easily measured surface indices. A common index is the random roughness of a surface - the standard deviation of point elevations within a defined area [10]. It has been shown, however, that one of the reasons that erosion and overland flow modelling results are still not satisfactory [11,12] is the lack of input data that capture the heterogeneity of the area under study. Therefore, there is a need for measurement methods that precisely characterise soil micro-topography, while allowing acquisition of data that is easy, fast and low-cost.

A wide variety of techniques exist for measuring soil surface roughness. These methods can be widely classified into two groups: contact and non-contact methods [13]. Pin meters [14,15] and roller chain measurements [16] are contact methods while non-contact methods include photogrammetric [17,18], infrared [19], ultrasonic [20], laser techniques [21,22], interferometry [23], and satellite radar measurements [24].

Contact instruments for topography characterisation are normally inexpensive and simple to use. However, they are affected by poor performances in terms of data density (often just in a single profile with few points). Additionally, they can deform the soil surface they are intended to measure, particularly on soft, loose grained or wet soils. Finally, mostly of these methods are quite labour-intensive.

Non-contact instruments, such as optical profilometers based on laser scanning [21] or digital photogrammetry provide high density three-dimensional data sets and fast non-destructive scanning [25]. However, there are also a number of weakness associated with optical instrumentation. The primary challenge is its high complexity and considerable capital costs (e.g. laser scanning), although McKenzie et al. [23] demonstrate a very simple technique based on interferometry. Other problems include: electronic noise due to intense sun light; signal loss or worsening in the presence of dust or smoke in the measurement domain; requirement for fast computers and substantial expertise; and long processing times. These challenges have limited their application in the field of hydrology.

“Structured light” is the method of projecting known light patterns (i.e. structures) onto an uneven scene and capturing the changes in the pattern caused by the surface. Those changes are analysed to ‘back calculate’ the 3D geometry of the surface. See Geng [26] for a full description of structured light and its uses. Modern structured light cameras are generally relatively inexpensive, highly portable (handheld sizing), and easy to use with minimal setup. The technology is currently used for applications as diverse as computer vision [27], medical scanning [28,29], and biometric security on mobile phones (https://support.apple.com/en-us/HT208108). We propose that this range of uses might extend to characterisation of the three-dimensional micro-topography of soil surfaces. Here we report on the usage of a 3D camera that uses structured light for this purpose.

## Methods and materials

### The 3D camera

**Fig. 1:**
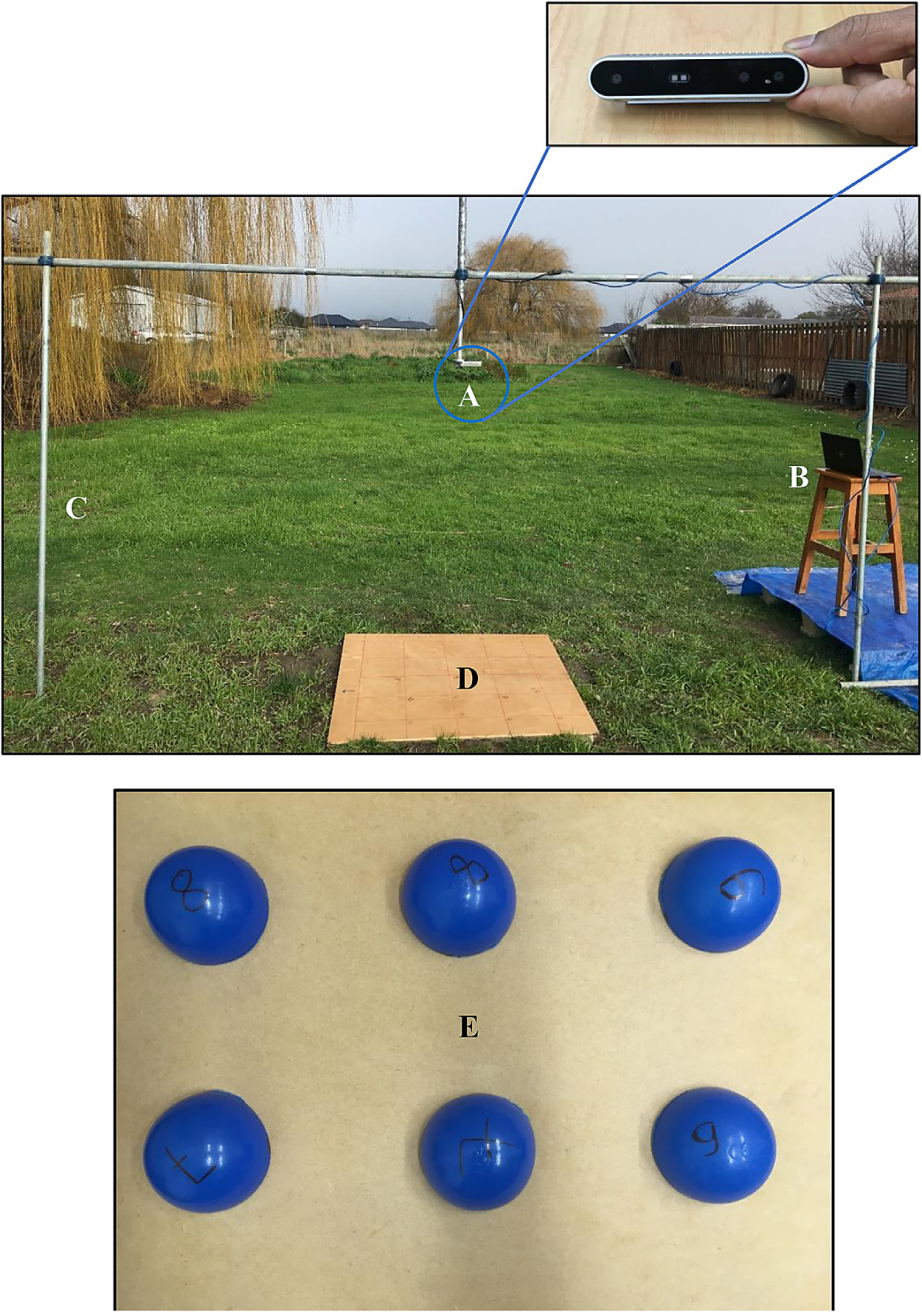
Setup consisting of (A) Intel RealSense Depth Camera, (B) Laptop, (C) Support legs, (D) Simulated smooth soil surface (plywood), and (E) Simulated rough soil surface using plastic hemispheres.

The 3D camera used was an Intel RealSense Depth camera (Model D415; www.intelrealsense.com/depth-camera-d415; henceforth referred to as the 3D camera). The camera was suspended from an aluminium frame using a tripod mount. The frame was constructed with adjustable legs to allow variation in camera heights over the soil surface (Fig. 1).

The 3D camera is a structured light depth camera made up of several components which all target the same field of view and contribute to creating the final 3D point cloud. Both visible and infrared radiation (IR) data are collected by the camera. The camera's IR emitter is used to project a structured IR pattern on to the soil surface. This is used in conjunction with a visual light receiver and two IR sensors paired in stereo to capture both IR and visible light information from the camera's overall field of view. Depth information is produced by correlating the points captured by each of the three image sensors to map differences and calculate a relative depth value for any given point. Depth values are given as a distance from the camera to the surface.

The camera can capture data at a 1280 × 720 pixel resolution in a range from 0.45 m up to approximately 10 m distance from a surface with the resolution and precision of the results varying depending on calibration, camera to object distance, nature of target objects in the scene, and lighting conditions. The manufacturer-specified measurement tolerance is ≤2% of the distance to the surface. This means at a camera height of 1 m, based on manufacturers specifications, we can expect the camera to be accurate to at least 0.02 m. Our findings (see Section 3.2) suggest that this estimate of precision by the manufacturer may be conservative, with the actual measurement tolerance being much better than advertised. The use of stereoscopic image sensors sets this camera apart from single-sensor options by providing greater depth accuracy than single-sensor models. The camera is lightweight (72 g) and small (99 mm × 23 mm × 20 mm).

For image acquisition, the 3D camera is connected to a computer using a USB 3.1 cable with the Intel RealSense Viewer software (https://www.intelrealsense.com/developers/) used to capture images and set various image and post-processing options. The camera is positioned and aligned with the area of interest (defined surface of soil) using the visible-light sensors and then the view mode is switched to 3D mode. For more details of the procedure see the Supplementary Material. As an alternative to the supplied Viewer application, Intel also provides a comprehensive software development kit (SDK) that can be used to access and fine-tune many aspects of the camera's capabilities. We used Intel's Python wrapper for the SDK to automate the image capture process, creating a script to capture 3D point clouds of the soil surface at defined intervals. This automation allowed us to track changes in the soil surface topography over time. Additionally, use of the SDK tools provides more control over post-processing options and image tuning than the options available in the Viewer application.

The 3D camera has a frame rate of 30 frames per second when the sensors are active. To address the practicalities of acquiring multiple sequential images, we wrote a Python script that captured a single frame at user-set time intervals. The sequence used was: initializing the camera sensor at the chosen interval; waiting for five frames to allow the camera's sensors to adjust for exposure; and then capturing the next frame in the sequence; before disabling the camera's sensors and clearing the frame buffer to optimize computing resources on the connected laptop.

The camera arrives pre-calibrated out of the box but can be re-calibrated if needed. We calibrated the camera following the factory-supplied calibration instructions and parameters.

### Assessing the performance of the 3D camera

The ability of the 3D camera to acquire elevation data accurately was tested on a variety of surfaces. In order to estimate accuracy and precision, the camera was suspended from an aluminium frame and tripod mount that allowed for a range of camera heights above the surface (Fig. 1). Starting from 1500 mm, images were taken at six heights down to 750 mm. For this trial we used smooth flat plywood (400 mm × 400 mm) placed centrally in the camera's field of view to simulate a surface with near-zero roughness. The camera and the plywood were levelled horizontally using a spirit level. The average distance of the set of points from the camera within the 400 mm × 400 mm target region at each height were used to quantify the effect of distance on accuracy of the camera by comparing against distance measured using a tape measure (accuracy to ±0.05 mm). For the precision test, the distance to the plywood was set to 1055 mm and distance was measured repetitively (*N* = 49). The variation of the repeated measurements was used to characterise the precision of the 3D camera.

Additionally, in order to evaluate the camera's ability to accurately determine surface area of a non-plane surface, artificial models were built using plastic hemispheres (Fig. 1). Six artificial models were built with an increasing number of 60 mm-diameter hemispheres (0–6 hemispheres) placed on the plywood, with 3D images taken at varying heights as described above. To evaluate the accuracy of surface area estimation, linear regression was fitted between the measured surfaces area and the theoretical surface area (cf. Gilliot et al. [17]).

To further characterise the reproducibility for capturing soil micro-topography data, 3D images were acquired on soil surfaces of varying roughness that had little slope. These images were from a height of 1000 mm. For each of the soil surfaces, the 3D camera measurements were repeated 20 times. Random roughness of the surface was calculated as the standard deviation of the slope-corrected elevation measurements for each of the 20 repeated images (c.f. Polyakov and Nearing [21]).

## Results and discussion

### Informal testing

In informal preliminary testing, we found that the camera was able to acquire data from a wide variety of artificial and natural objects. It performed well in a variety of lighting conditions, however we noted some limitations. The camera relies upon both visible and IR light to create the point cloud, so adequate lighting conditions are required to produce a high-quality depth map. The camera did not work well in conditions with lower lighting levels such as might be encountered indoors or in dim light outdoors. The camera was also challenged by the presence of some forms of artificial light, such as fluorescent lighting, which interfered with the IR sensors. Performance was also unreliable in very bright outdoor light when the surface reflected light directly at the camera. Finally, the camera's performance was occasionally affected by the presence of shadows cast across the field creating false depth information. Note that this only applied to shadows of external origin (e.g. trees, buildings, people) not the internal shadows that arise from a rough soil surface. When these effects arose, they were obvious in the point cloud with phantom surfaces substantially above or below the real surface and so could be removed in post-processing.

In this preliminary testing we found it was important to ensure the camera was fixed level and we ensured this using a spirit level. As the camera was mounted using a single standard tripod mount, we found that it could be difficult to maintain the levelling of the camera on occasion. This could be mitigated with a different style of mounting or through vigilant attention to levelling throughout usage.

The camera produced a lower areal density of data points when used on sloped surfaces, with the density of data points decreasing as slope increased. This is because the sloped surface reflects IR signals away from the IR sensor resulting in a reduced density of information and perhaps some blank spots. This limitation means that the accuracy may be lower than we report when used on surfaces with a noticeable degree of slope. While this effect may be possible to mitigate by mounting the camera normal to the surface, doing so in practice introduces complications to the camera mounting method. Varying degrees of slope on a surface may further complicate any attempt to mount the camera at true normal to the surface.

The methods chosen for post-processing images, either in the camera software or through the SDK, also affected the quality of data generated. While there are post-processing options (spatial filter, decimation filter, disparity transform, temporal filter, and hole filling) designed to improve the overall quality of the 3D image, the different techniques can result in a reduction of the final number of depth points collected (decimation filter, temporal filter) or artificially smooth out genuine roughness of the soil surface (disparity transform, spatial filter, hole-filling). For these reasons, understanding the effects of the different post-processing filters is important for making informed decisions about the trade-offs of using the techniques compared to taking a more manual approach to image cleaning and post processing after the images are captured.

#### Depth camera performance

**Fig. 2:**
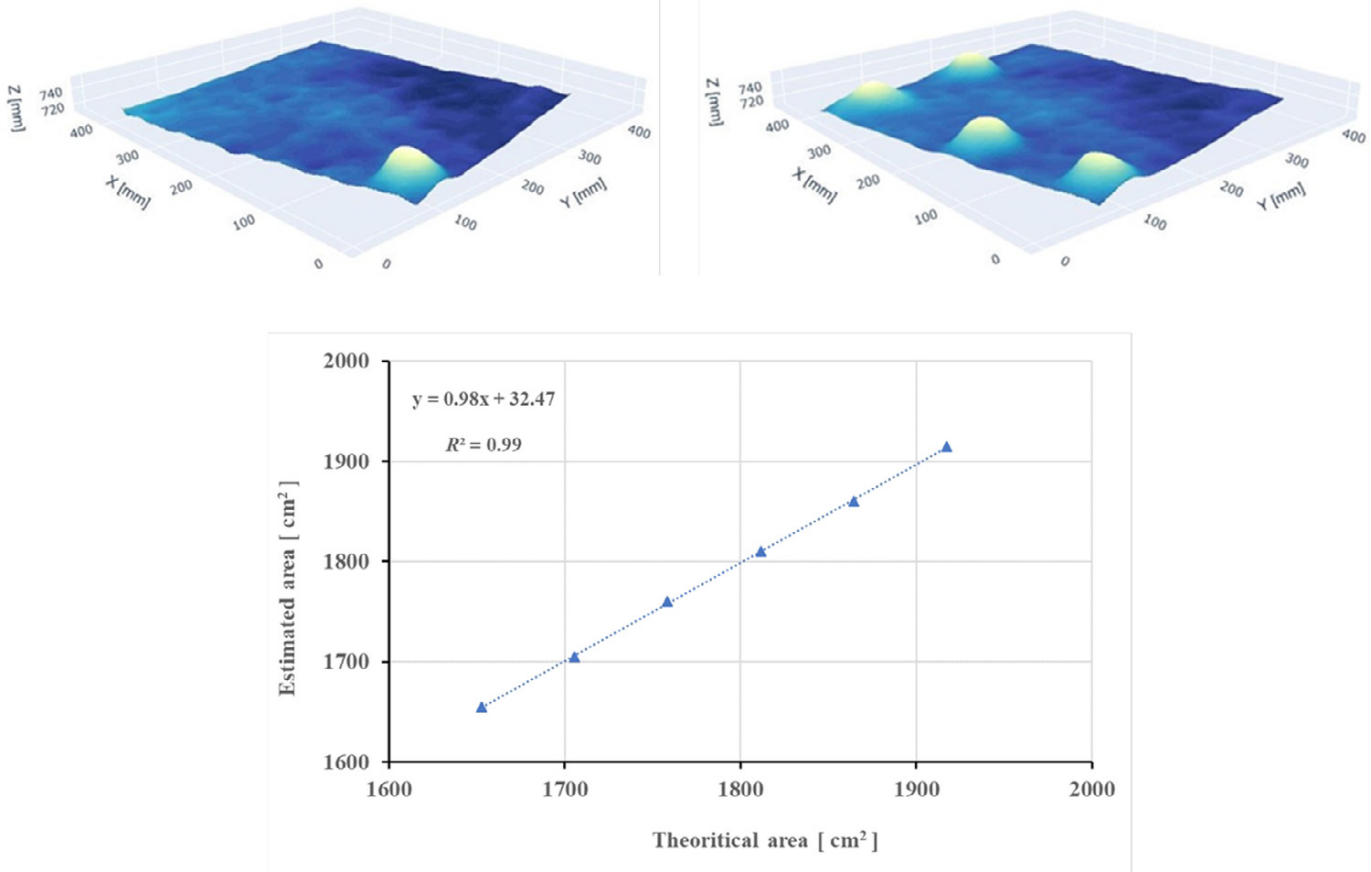
Accuracy of the surfaces are estimation: (a) two of the six 3D models calculated, and (b) linear regression between the theoretical model's area and the estimated one using the 3D camera at camera-surface distance of 750 mm.

Results of the first accuracy test performed on a 400 mm by 400 mm smooth soil surface simulated with flat plywood are presented in Table 1. The number of points measured decreased with distance as the plywood occupied ever smaller proportions of the camera's field of view, and so always was less than the 921,600 points (1280 × 720) that comprise the full field. Accuracy was calculated as the deviation of the average distance measured by the camera from that measured independently using a tape measure. Absolute accuracy generally decreased with increasing height of the camera. At a height of 750 mm the relative accuracy was 0.3% while at 1500 mm accuracy was −0.5%. Precision of the instrument is defined as the closeness of measurements to each other. In the repeated measurements at 1055 mm (*N* = 49), 95% of the repeat measurements were ±1 mm from the mean value. Similarly, the slope of regression line between predicted and measured surface areas was close to 1 (0.99) at camera-plywood distance of 750 mm (Fig. 2). The average error of the six surface estimations varied from 0.56% (750 mm) to 2.3% (1500 mm).

**Table 1:** Accuracy acquired on flat plywood surface of 400 mm by 400 mm from six camera heights above the surface. The data presented are: the number of points captured in the measurement region (*N*); the average and standard deviation of distance between the camera and the surface as measured by the camera (*D*); and accuracy (*A*) as the deviation between *D* and that measured independently.

	Height of the camera above surface (mm)					
	750	900	1055	1204	1352	1500
*N*	63,600	44,300	32,400	24,900	19,700	16,000
*D* (mm)	752 ± 2	899 ± 2	1051 ± 4	1200 ± 4	1346 ± 6	1493 ± 6
*A* (%)	0.3	−0.1	−0.4	−0.3	−0.4	−0.5

**Fig. 3:**
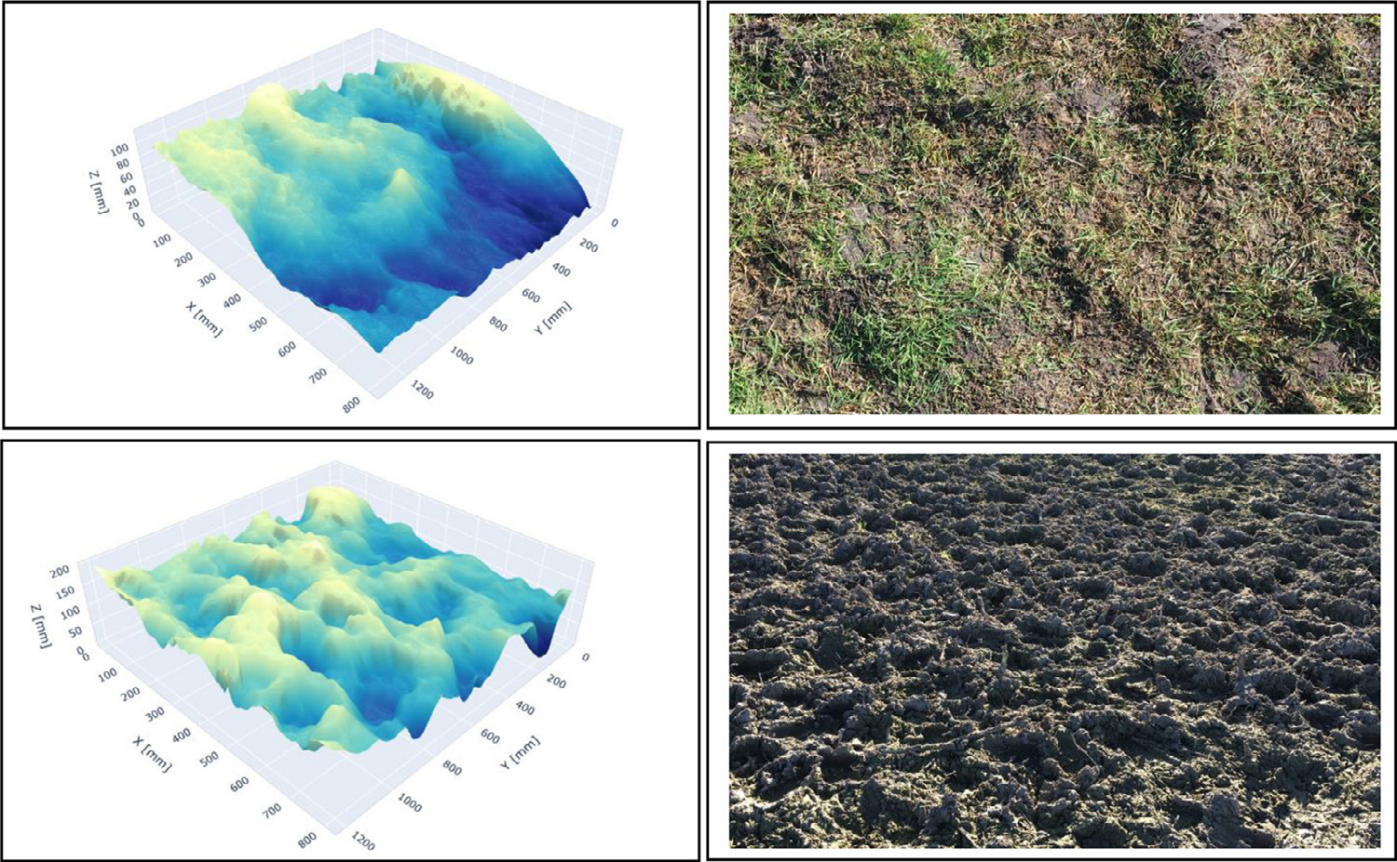
Digital elevation model (DEM; 1 mm × 1 mm) for the intact (upper) and damaged (lower) soil surfaces generated using the 3D camera ~1000 mm above the surfaces to capture an area of 1200 mm × 800 mm. The processing methods described in the Supplementary Material. DEMs for each soil type are shown on the left and corresponding example photographs of the respective soil types are shown on the right.

In order to test the ability of the 3D camera to reconstruct soil micro-topographies, a series of measurements was carried out on two soils with varying surface conditions. Prior to measurement, any vegetation and straw present were removed from the soil surface without disturbance. Fig. 3 shows a 3D rendering, at a 1 mm × 1 mm spatial resolution, of two dairy pasture soils and corresponding example photographs of each soil. The first soil surface (labelled as ‘intact’) is a pasture that has been grazed close to the ground but is otherwise undamaged soil, while the second soil surface has been damaged by hoof action (labelled as ‘damaged’). The images show that the 3D rendering is able to clearly reproduce the visible features and allow detection of the micro-topography. Moreover, good reproducibility of the 3D image results (roughness and elevation) on both soil surfaces was achieved (Table 2). Across 20 repetitions, the average and standard deviation of the elevation of intact soil was measured by the camera at 1061 ± 0.40 mm and at 1074 ± 0.28 mm for the damaged soil. The low standard deviations indicate repeat measurements mostly return the same elevation points. Random roughness of these surfaces was estimated at 13.8 ± 0.02 mm for the intact soil and 26.0 ± 0.04 mm for the damaged soil. These measurements show high precision (i.e. they are highly repeatable).

**Table 2:** Repeatability acquired on two soil surfaces (see Fig. 1) when the 3D camera was about 1000 mm above the soil surface and the measurement area was 1200 mm by 800 mm. The data presented are: the number of repetitions, the number of points captured in the measurement region (*N*); the average and standard deviation of distance between the camera and the surface as measured by the camera (*D*); the maximum (*D_max_*) and minimum (*D_min_*) of the points in each of the images and their standard deviations; and the random roughness (*R*) for each soil surface and its standard deviation.

	Intact soil	Damaged soil
Repetitions	20	20
*N*	196,440	190,200
*D* (mm)	1074 ± 0.28	1061 ± 0.40
*D_max_* (mm)	1129 ± 0.70	1179 ± 1.80
*D_min_* (mm)	1015 ± 0.51	984 ± 2.00
*R* (mm)	13.8 ± 0.02	26.0 ± 0.04

## Conclusions

We have summarised the use of a simple, commercially-available, structured light camera as a cost-effective solution for three-dimensional characterisation of soil micro-topography. The camera is relatively cheap and can be implemented quickly without expert knowledge. The performance of camera was analysed and was demonstrated to give good accuracy and precision. We found that it worked reliably on a wide variety of natural materials and could be used rapidly over a variety of soil surfaces. It required only one operator.

The main limitation of the camera was concerned with the quality of the lighting conditions. To mitigate the impacts of lighting conditions on the data quality, we recommend operating the camera outdoors, avoiding external shadows being cast on the surface, and avoiding direct sunlight being reflected towards the camera's sensors. Examination of the point cloud will clearly show the effects of poor lighting conditions. Other methodological concerns are a secure mounting to keep the camera level, care when using on heavily sloped surfaces, and ensuring that post-processing software is only utilised where appropriate, so image quality does not unnecessarily become overly degraded.

## Appendix Supplementary materials

Image application 1
