# Supplementary material for: Characterisation of soil micro-topography using a depth camera
Source: MethodsX. 2020 Nov 12;7:101144. doi: 10.1016/j.mex.2020.101144 (PMC7724195; doi:10.1016/j.mex.2020.101144)
Supplement: Image [file mmc1.docx]

# **Supplementary material - Characterisation of soil micro-topography using a depth camera**

Laura Grundy, Chandra Prasad Ghimire ^*^, Val Snow

AgResearch, Private Bag 4749, Christchurch 8140, New Zealand

^*^Corresponding author:

Chandra Prasad Ghimire ([chandra.ghimire@agreresearch.co.nz](mailto:chandra.ghimire@agreresearch.co.nz))

Private Bag 4749

Christchurch 8140

New Zealand

Phone: +64 33 21 88 31

## *S1. Collect 3D Images*

The first step in generating the DEM is capturing the 3D point cloud data relating to the soil surface. The 3D camera is connected to a computer using a USB 3.1 cable. To capture the images, the Intel RealSenseViewer software can be used on the computer. This software is available to download from the Intel website, does not require any installation and runs on the Windows operating system. After running the RealSense Viewer Application, the user is presented with options to activate different aspects of the camera via toggle switches on the left-hand side of the program. We found the most useful settings were to activate both the RGB and depth camera components as well as enabling the post-processing toggle for the depth module to improve the quality of the captured image. Once the camera settings have been configured and the positioning of the camera aligned to capture the area of interested (plot/defined surface of soil), the view mode can be switched to 3D using the controls in the top right. When ready, the save icon in the top right can be used to capture a 3D point cloud of the current viewpoint.

As an alternative to the 3D Viewer application, Intel also provides a comprehensive SDK that can be used to access and fine tune many aspects of the camera’s capabilities. We found success in using the supplied Python wrapper for the SDK to automate the image capture process, creating a script to capture 3D point clouds of the view at defined intervals to track changes in the soil surface topography over time. The SDK provides further control over post-processing options and image tuning.

## *S2. Inspect, clean, and export the 3D point cloud*

Once satisfactory 3D images representative of the area had been collected, the 3D point clouds were loaded into MeshLab to convert into a format suitable for loading into the GIS software as a text delimited layer. MeshLab also provides the ability to inspect and clean the 3D point cloud to produce higher quality DEMs. Images taken by the 3D camera are commonly exported in a Polygon File (ply) format. We loaded these into MeshLab using the Import mesh function. The 3D point cloud can then be refined using the point selection and point deletion tools to crop the mesh to the area of interest and to remove any points in the 3D space that are outliers caused by noise or interference with the camera’s light detection system.

MeshLab also allows the cleaned 3D point cloud to be converted and exported as xyz format. This is useful as it provides a simple list of all points that make up the cloud in a format that is easily imported into GIS software and thus can be used for further processing of the 3D image. The camera by default exports images into ply format. This format is not readily used in applications outside of the 3D imaging domain and cannot be readily inspected without specific software. MeshLab, being free and opensource is a valuable tool for converting the ply files into the more versatile xyz format.

## *S3. Create the DEM using QGIS and B-spline Interpolation*

After point clouds have been suitably cleaned and exported to xyz format, they can then be loaded into the geographical information software. We used QGIS. This is most easily done by first loading the xyz files in as new layers in the project. By selecting “add new text delimited layer”, the user is then able to load in the xyz file, giving the x values as field 1 from the file and the z value as field 2. The appropriate coordinate system for the data must also be selected to ensure that the resulting DEM is calculated at the correct scale and dimensions. Once the point cloud is loaded, a reconstruction of the surface can be created using Multi-level B-spline Interpolation. This technique is preferred as it offers a high degree of fidelity in the reconstructed surface and performs well with irregular point cloud data sets similar in nature to those produced by the 3D camera [1]. We found that using the algorithm without B-spline refinement produced sufficient quality surface reconstructions for further analysis.

The B-Spline Interpolation produces a raster in SDAT format which can be imported and used as is in QGIS to do further analysis or imported into other software such as ARCGIS or processed using python.

## *S4 References*

[30] Lee S, Wolberg G, Shin SY. Scattered data interpolation with multilevel B-splines. IEEE Transactions on Visualization and Computer Graphics 1997;3(3):228-44. <https://doi.org/10.1109/2945.620490>.
